# Novel model based on ultrasound predict axillary lymph node metastasis in breast cancer

**DOI:** 10.1186/s12880-023-01090-7

**Published:** 2023-09-18

**Authors:** Biyu Zheng, Qingshuang Chen

**Affiliations:** Department of Ultrasound, The First Affiliated Hospital of Xiamen University, School of Medicine, No. 55, Zhenhai Road, Siming District, Xiamen, Fujian China

**Keywords:** Ultrasound, Breast cancer, Axillary lymph, Metastasis, Predict

## Abstract

**Background:**

Whether there is axillary lymph node metastasis is crucial for formulating the treatment plan for breast cancer. Currently, invasive methods are still used for preoperative evaluation of lymph nodes. If non-invasive preoperative evaluation can be achieved, it will effectively improve the treatment plan.

**Objective:**

Constructed a predict model based on ultrasound examination, which forest axillary lymph node metastasis in breast cancer, and validated this model.

**Method:**

Patients admitted to Xiamen First Hospital from April 2018 to August 2021 with complete case data were included in this study. Patients who had undergone breast cancer resection and axillary lymph node dissection or sentinel lymph node biopsy were divided into a training and validation cohort in a 7:3 ratio. In the training cohort, patients were divided into metastatic and non-metastatic groups based on whether axillary lymph nodes had metastasis. The parameters of the two groups were compared, and statistically significant parameters were included in multivariate analysis. Then, a Nomogram model was constructed, named Lymph metastasis predict model (LMPM). Calibration curves, receiver operating curve (ROC), and decision curve analysis (DCA) were plotted between the training and validation cohort, calculate the risk score of each patient, identify the optimal cutoff value, and test the predictive efficacy of LMPM.

**Result:**

Two hundred seventy-three patients were enrolled in final study, the average age 49.7 ± 8.7, training cohort included 191 patients, the diameter of breast cancer, the lymph node peak systolic flow velocity (LNPS) and the cortex area hilum ratio (CH) of lymph node were exist significant difference in metastatic and non-metastatic group. Multivariate analysis showed cancer diameter, LNPS and CH included in LMPM, the cutoff value was 95, the calibration curve, ROC, DCA in training and validation cohort show satisfactory result.

**Conclusion:**

The predict model-LMPM, can predict axillary lymph node metastasis in breast cancer, which is useful for developing personalized treatment plans. However, further validation of the model is required by incorporating a larger number of patients.

## Introduction

Breast cancer, alone accounting for 30% of female cancer, is a prevalent malignancy affecting women worldwide [1], with axillary lymph node(ALN) metastasis being a crucial factor in staging and treatment decisions [2], if axillary lymph node metastasis is confirmed, axillary lymph node dissection is likely to be adopted. Therefore, the early diagnosis of axillary lymph node metastasis in patients is of significant importance. Although sentinel lymph node biopsy holds important value, however, it cannot be performed on all axillary lymph nodes, and this procedure carries the risk of complications, including axillary vein thrombosis, injury to the axillary motor nerves, lymphedema affecting the arm and breast, the formation of seroma, and impairment of shoulder function. Minimizing these avoidable complications in patients without lymph node metastases is an ongoing research focus. The development of noninvasive techniques for predicting lymph node status holds universal advantages in improving patient care.

With advancements in medical technology, non-invasive prediction of lymph node metastasis has become an urgent task in contemporary medicine. Several methods have been developed to predict whether axillary lymph nodes are malignantly invaded non-invasively. Yu included enhanced MRI images of early-stage breast cancer patients from four centers. They utilized a semi-automatic method to delineate tumor regions on the images and subsequently extracted the grayscale information from these areas. A total of 863 dimensional features were extracted. Using an unsupervised learning approach, they classified the tumors and then further selected significant indicators for prediction. This led to the construction of a Nomogram model. The model with an AUC exceeding 90% in both modeling and validation cohorts [3]. Radiomics, as one of the most rapidly developing disciplines in medical imaging, has significant guiding implications for cancer diagnosis and prognosis. Additionally, many researchers have combined machine learning with radiomics to construct models for predicting axillary lymph node metastasis status. A meta-analysis involving 1618 breast cancer patients showed an AUC of 89%, but this technique remains limited to the research stage due to its complex operational skills [4] .

Breast ultrasound, as a routine screening method for breast cancer, holds paramount importance for clinical practitioners. plays a vital role in the evaluation of breast cancer, providing essential information for predicting axillary lymph node involvement. This comprehensive study aims to consolidate the current understanding of ultrasound characteristics associated with axillary lymph node metastasis in breast cancer, emphasizing the significance of ultrasound in clinical decision-making and patient management. We discuss various ultrasound features, including size, shape, echogenicity, cortical thickness, and vascularity. Through an in-depth analysis of the data, we provide breast ultrasound experts with an updated understanding of the ultrasound characteristics associated with axillary lymph node metastasis in breast cancer, facilitating accurate diagnosis, staging, and treatment planning. Two-dimensional ultrasound allows observation of tumor size, morphology, and internal features, such as deep growth and the presence of calcifications. Concurrently, ultrasound facilitates the convenient observation of axillary lymph node status, including the assessment of morphology, cortex-to-medulla ratio, disappearance of lymphatic sinuses, and color Doppler flow signals between the tumor and lymph nodes. Various hemodynamic parameters, such as peak systolic flow velocity (PS), end diastolic flow velocity (EDF), systolic velocity to end of diastolic velocity ratio (S:D), resistance index (RI), and pulsatility index (PI), can also be measured. Compared to methods like MRI and radiomics, conventional ultrasound is characterized by its non-radiation, repeatability, and cost-effectiveness. Some scholars have previously constructed predictive models using ultrasound examination features and clinical characteristics. However, the models they developed were not intuitive and not suitable for direct clinical application [5]. Guo combined ultrasound radiomics with machine learning to create a predictive model for axillary lymph node metastasis, which showed high sensitivity [6].

To enhance the diversity of choices for clinical practitioners and patients, we employed conventional ultrasound to build a predictive model. In this study, we utilized conventional ultrasound examination parameters to construct a non-invasive predictive model for axillary lymph node metastasis, named Lymph metastasis predict model(LMPM), and validated the model’s predictive performance. The model was developed using a training cohort comprising 191 patients and subsequently validated using an additional set of 82 patients from the same institution. To visually represent the model, a nomogram was created, and to facilitate its utilization in a clinical setting, which offers comprehensive insight into the model’s parameters.

## Patients and method

### Patients

**Fig. 1:**
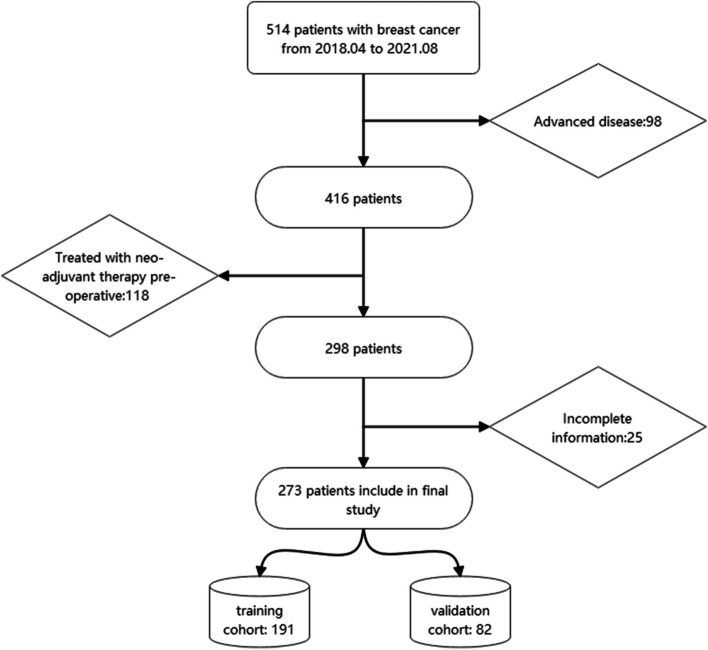
Patient inclusion flow

This study was approved by Ethics Committee of Xiamen First Hospital, all patients signed informed consent forms. This study enrolled a consecutive series of 273 patients with primary invasive early breast carcinoma treated at the Xiamen First Hospital from 2018.04 to 2021.08 were enrolled in this study. All patients were divided into training and validation cohort in a ratio 7:3 randomly. The patients inclusion flow was showed in Fig. 1.

The eligible participants for this study consisted of female patients diagnosed with early invasive breast cancer, as per the clinical TNM stage outlined in the 8th edition of the American Joint Committee on Cancer (AJCC) Cancer Staging Manual. Specifically [7], patients with stages T1-3 and N0-1 were included. Additionally, the inclusion criteria required the patients to have positive axillary ultrasound findings, defined as the presence of at least one lymph node visible by ultrasound. The participants must have undergone either a successful sentinel lymph node biopsy (SLNB) or Axillary lymph node dissection (ALND). Patients with locally advanced disease (TNM stage T4 or N2-3), those who received neo-adjuvant treatment, or those with bilateral breast cancer, or lack of insufficient information were excluded from the study.

### Ultrasound

**Fig. 2:**
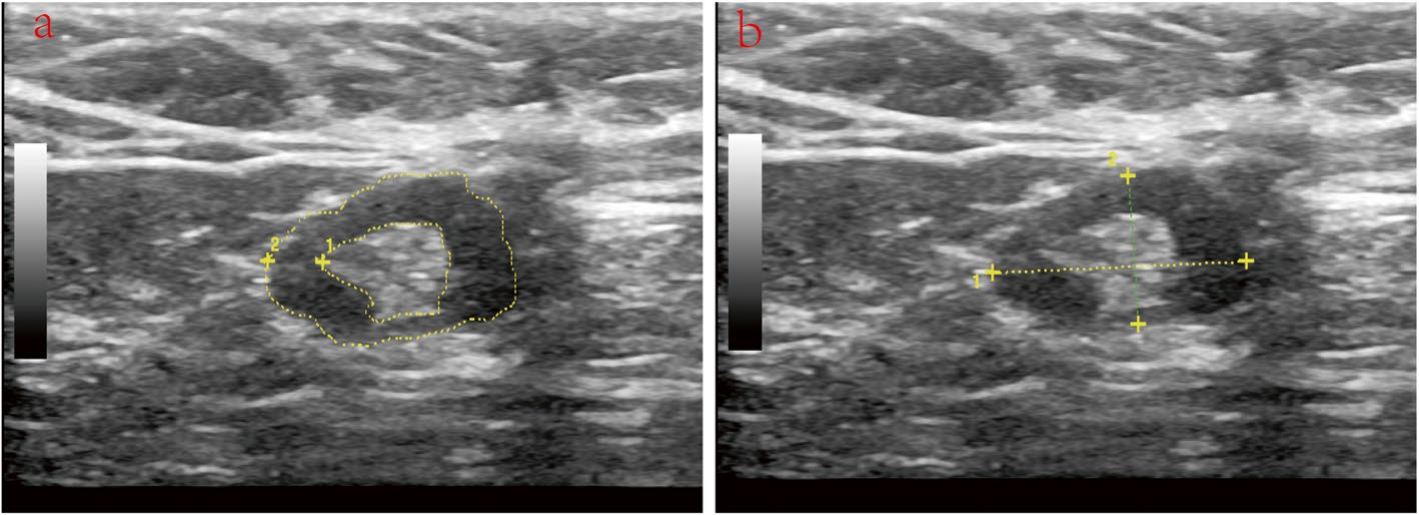
Lymph Node Measurement Diagram: **a** Measurement of the longitudinal and transverse diameters of lymph nodes. **b **Measurement of the cortical area and hilum area of lymph nodes

All breast ultrasound examinations were conducted using a Philips unit equipped with a 5-12-MHz linear-array broadband transducer (EPIQ7). To ensure consistency, the evaluation process adhered to standardized protocols utilizing the same ultrasound machine. Bilateral breasts were thoroughly assessed to detect any presence of multifocal or multicentric breast cancer. Characteristics of breast cancer and lymph nodes, including number of lesions and nodes, transverse diameter, longitudinal diameter, longitudinal-to-transverse(LT) axis ratio, cortical thickness. Initially, grayscale imaging was employed for lymph node detection. In cases where image contrast required improvement, spatial compound or tissue harmonic imaging techniques were utilized. If an enlarging lymph node was identified during ultrasound examination, we captured an image at the section displaying the maximum lymph node size. On the ultrasound monitor, we carefully traced the hypoechoic cortical portion and hyperechoic hilar portion(CH), subsequently measuring their respective areas (in cm^2^) at the section with the maximum lymph node size, calculate their ratio. (Fig. 2a). Additionally, we measured the longitudinal and transverse diameters of the lymph node (in mm) and calculated the LT axis ratio by dividing the longitudinal diameter by the transverse diameter (Fig. 2b).

Two experienced radiologist (Biyu Zheng, Qingshuang Chen), who possesses over a decade of expertise in breast ultrasound imaging, thoroughly examined and together reevaluated the ultrasound results. For palpable tumors, a Doppler frequency of 5-12 MHz linear-array broadband transducer was employed, capturing vascular information from different angles. To enhance the detection of slow flow, the filter was set at 50 Hz, and the pulse-repetition frequency (PRF) was adjusted to prevent aliasing. The color Doppler gain was gradually increased until background noise became apparent, followed by a slight reduction to eliminate noise while maintaining optimal flow sensitivity. Doppler angle correction was consistently performed using the machine’s built-in capabilities. In the Doppler image, a cancer vessel was identified based on the constant and persistent presence of color flow within the cancerous area in one specific plane. Another vessel was considered separate if it disappeared and reappeared during continuous scanning of the breast mass. Doppler arterial waves with regular form and rhythm were required for flow indices measurement. Tumor and lympha’s parameters such as peak systolic flow velocity (PS), end diastolic flow velocity (EDF), systolic velocity to end of diastolic velocity ratio (S:D), resistance index (RI), and pulsatility index (PI) were recorded from the Doppler frequency spectra. We recorded lymph node peak systolic flow velocity as LNPS, lymph node end diastolic flow velocity as LNEDF.

### Surgical

The primary disease was treated through surgical intervention, which entailed either breast-conserving surgery or mastectomy, with or without the option of breast reconstruction. Systemic and radiation therapy were administered in accordance with the clinical guidelines established locally.

### Pathology

Tumor tissues were acquired from specimens embedded in paraffin for staining with hematoxylin and eosin (H&E), immunohistochemistry (IHC), and fluorescence in situ hybridization (FISH). The grading of invasive tumors was assessed based on the Nottingham (Elston-Ellis) modification of the Scarf-Bloom-Richardson grading system. Following the surgery, all lymph nodes were examined through serial section H&E staining. In cases where no cancer cells were detected on H&E staining, IHC staining was conducted to determine the presence of micrometastasis (0.2–2 mm cancer foci). Immunostaining for ER, PR, P53, and VEGF-C was considered positive if observed in over 10% of tumor cells. Her-2 positivity was defined as a score of 3 + on IHC or amplification on FISH. Ki-67 positivity was characterized by nuclear staining of tumor cells exceeding 14%, while negativity was assigned to cases where it was equal to or below 14%.

### Statistical

The patients were divided into training and testing cohorts in a random manner, maintaining a ratio of 7:3. Categorical variables were presented as percentages and compared using chi-square tests. For continuous variables, first, the normality of the data is checked using the Shapiro-Wilk test, and the homogeneity of variances is assessed using Levene’s test. If both assumptions are met, an independent two-sample t-test (student’s t-test, two-sided) is used. The inclusion of predictors followed three guiding principles: previous literature findings, disparities observed in univariate analysis, and clinical significance.

To ensure the absence of collinearity, all variables were assessed using a correlation matrix.

We divided the patients in the modeling cohort into two groups, the metastasis group and the non-metastasis group, based on whether axillary lymph node metastasis occurred. In the univariate analysis stage, appropriate statistical methods were used to compare various parameters between the two groups. Subsequently, indicators with statistically significant differences (*P* < 0.05) and potential predictors of lymph node metastasis were included in the binary logistic multivariate analysis. After the multivariate analysis, indicators independently predictive of axillary lymph node metastasis were incorporated into the final predictive model. A nomogram was developed as a graphical representation of the model. To assess the calibration of the model, the patients score were calculated. To determine the optimal cut-off value for clinical use, Youden index was utilized. Calibration was evaluated graphically. The predictive model was further validated using a validation cohort. The receiver operating characteristic (ROC) curve was plotted, and the area under the curve (AUC) was employed to evaluate the predictive accuracy of the model. Delong test was used for compared the AUC of different parameter. The decision curve analysis (DCA) was plotted. All tests were two-sided, and a *p*-value of less than 0.05 was considered statistically significant. Statistical analyses were performed using SPSS software (version 26, SPSS Inc., Chicago, IL) and “R” software (version 3.6.5).

## Result

### Basic information of training and validation cohort

This study enrolled 273 patients, 119(43.6%) patients suffered with lymph metastasis, the average age was 49.7 ± 8.7 years, all of the parameters are equal in training and validation cohort, the result showed in Table 1.

**Table 1:** Basic characteristics of all patients

	Total	Train	Test	*P*-value
Lymph metastasis				
No	154 (56.4%)	105 (55.0%)	49 (59.8%)	0.51
Yes	119 (43.6%)	86 (45.0%)	33 (40.2%)	
Menopausal				
Premenopausal	131 (48.0%)	88 (46.1%)	43 (52.4%)	0.36
Postmenopausal	142 (52.0%)	103 (53.9%)	39 (47.6%)	
Location				
Upper-out	74 (27.1%)	54 (28.3%)	20 (24.4%)	0.97
Lower-out	34 (12.5%)	24 (12.6%)	10 (12.2%)	
Upper inner	69 (25.3%)	47 (24.6%)	22 (26.8%)	
Lower inner	76 (27.8%)	52 (27.2%)	24 (29.3%)	
Center	20 (7.3%)	14 (7.3%)	6 (7.3%)	
Histological grade				
I	54 (19.8%)	35 (18.3%)	19 (23.2%)	0.4
II	81 (29.7%)	61 (31.9%)	20 (24.4%)	
III	138 (50.5%)	95 (49.7%)	43 (52.4%)	
Histological type				
Ductal	245 (89.7%)	177 (92.7%)	68 (82.9%)	0.028
Lobular	15 (5.5%)	9 (4.7%)	6 (7.3%)	
Other	13 (4.8%)	5 (2.6%)	8 (9.8%)	
ER				
1+	39 (14.3%)	29 (15.2%)	10 (12.2%)	0.78
2+	36 (13.2%)	27 (14.1%)	9 (11.0%)	
3+	100 (36.6%)	67 (35.1%)	33 (40.2%)	
Negative	98 (35.9%)	68 (35.6%)	30 (36.6%)	
PR				
1+	37 (13.6%)	27 (14.1%)	10 (12.2%)	0.88
2+	46 (16.8%)	31 (16.2%)	15 (18.3%)	
3+	64 (23.4%)	43 (22.5%)	21 (25.6%)	
Negative	126 (46.2%)	90 (47.1%)	36 (43.9%)	
Her2				
Negative	178 (65.2%)	124 (64.9%)	54 (65.9%)	1
Positive	95 (34.8%)	67 (35.1%)	28 (34.1%)	
Ki67				
Smaller14	50 (18.3%)	32 (16.8%)	18 (22.0%)	0.31
Bigger14	223 (81.7%)	159 (83.2%)	64 (78.0%)	
P53				
Negative	97 (35.5%)	69 (36.1%)	28 (34.1%)	0.78
Positive	176 (64.5%)	122 (63.9%)	54 (65.9%)	
VEGF-C				
Negative	70 (25.6%)	50 (26.2%)	20 (24.4%)	0.88
Positive	203 (74.4%)	141 (73.8%)	62 (75.6%)	
Regular shape				
Yes	142 (52.0%)	99 (51.8%)	43 (52.4%)	1
No	131 (48.0%)	92 (48.2%)	39 (47.6%)	
Boundary				
Clearly	133 (48.7%)	87 (45.5%)	46 (56.1%)	0.12
Obscure	140 (51.3%)	104 (54.5%)	36 (43.9%)	
Echo				
Homogeneous	132 (48.4%)	85 (44.5%)	47 (57.3%)	0.064
Heterogeneous	141 (51.6%)	106 (55.5%)	35 (42.7%)	
Calcification				
No	135 (49.5%)	95 (49.7%)	40 (48.8%)	0.9
Yes	138 (50.5%)	96 (50.3%)	42 (51.2%)	
Age(years)	49.7 ± 8.7	49.9 ± 8.9	49.3 ± 8.3	0.61
Diameter(cm)	14.5 ± 5.2	14.6 ± 5.2	14.1 ± 5.4	0.31
Tumor peak systolic flow velocity(PS)	38.1 ± 6.1	37.6 ± 6.0	39.0 ± 6.1	0.089
Tumor end diastolic flow velocity(EDF)	10.1 ± 2.2	10.2 ± 2.2	10.0 ± 2.1	0.53
Tumor systolic velocity to end of diastolic velocity ratio(SD)	4.0 ± 1.1	3.9 ± 1.1	4.1 ± 1.2	0.15
Tumor resistance index(RI)	0.6 ± 0.2	0.6 ± 0.2	0.6 ± 0.2	0.28
Tumor pulsatility index(PI)	1.8 ± 0.3	1.8 ± 0.3	1.8 ± 0.3	0.31
lymphatic transverse	5.9 ± 1.3	6.0 ± 1.2	5.8 ± 1.3	0.24
lymphatic longitudinal	11.6 ± 2.1	11.8 ± 2.1	11.3 ± 2.0	0.068
cortex area hilum ratio(CH)	1.9 ± 0.3	1.9 ± 0.3	1.9 ± 0.3	0.49
Lymph peak systolic flow velocity(PS)	17.4 ± 6.2	17.4 ± 6.2	17.3 ± 6.3	0.97
Lymph end diastolic flow velocity(EDF)	8.0 ± 2.1	7.9 ± 2.1	8.1 ± 2.2	0.33
Lymph systolic velocity to end of diastolic velocity ratio(SD)	3.2 ± 1.7	3.2 ± 1.8	3.2 ± 1.7	0.43
Lymph resistance index(RI)	0.6 ± 0.2	0.6 ± 0.2	0.6 ± 0.2	0.97
Lymph pulsatility index(PI)	1.7 ± 0.3	1.7 ± 0.3	1.7 ± 0.3	0.31

### Basic information in training cohort

One hundred ninety-one patients were assigned into training cohort, 86 patients were suffered with lymph metastasis, the average age was 49.9 ± 8.9, the proportion of P53 positive and VEGF-C positive in lymph metastasis group were higher than non-lymph metastasis group. The diameter of breast cancer, the CH value and the PS of lymph in lymph metastasis group were higher than non-lymph metastasis group, above mentioned parameters has significant difference(*P* < 0.05). The other parameters were equal in both group. The result showed in Table 2.

**Table 2:** Data in training cohort

	Total	No metastasis	Lymph metastasis	*P*-value
Menopausal				
Premenopausal	88 (46.1%)	49 (46.7%)	39 (45.3%)	0.88
Postmenopausal	103 (53.9%)	56 (53.3%)	47 (54.7%)	
Location				
Upper-out	54 (28.3%)	30 (28.6%)	24 (27.9%)	0.31
Lower-out	24 (12.6%)	12 (11.4%)	12 (14.0%)	
Upper inner	47 (24.6%)	31 (29.5%)	16 (18.6%)	
Lower inner	52 (27.2%)	27 (25.7%)	25 (29.1%)	
Center	14 (7.3%)	5 (4.8%)	9 (10.5%)	
Histological grade				
I	35 (18.3%)	18 (17.1%)	17 (19.8%)	0.86
II	61 (31.9%)	35 (33.3%)	26 (30.2%)	
III	95 (49.7%)	52 (49.5%)	43 (50.0%)	
Histological type				
Ductal	177 (92.7%)	96 (91.4%)	81 (94.2%)	0.57
Lobular	9 (4.7%)	5 (4.8%)	4 (4.7%)	
Other	5 (2.6%)	4 (3.8%)	1 (1.2%)	
ER				
1+	29 (15.2%)	19 (18.1%)	10 (11.6%)	0.58
2+	27 (14.1%)	15 (14.3%)	12 (14.0%)	
3+	67 (35.1%)	37 (35.2%)	30 (34.9%)	
Negative	68 (35.6%)	34 (32.4%)	34 (39.5%)	
PR				
1+	27 (14.1%)	19 (18.1%)	8 (9.3%)	0.075
2+	31 (16.2%)	18 (17.1%)	13 (15.1%)	
3+	43 (22.5%)	17 (16.2%)	26 (30.2%)	
Negative	90 (47.1%)	51 (48.6%)	39 (45.3%)	
Her2				
Negative	124 (64.9%)	69 (65.7%)	55 (64.0%)	0.88
Positive	67 (35.1%)	36 (34.3%)	31 (36.0%)	
Ki67				
<=14	32 (16.8%)	20 (19.0%)	12 (14.0%)	0.44
>14	159 (83.2%)	85 (81.0%)	74 (86.0%)	
P53				
Negative	69 (36.1%)	45 (42.9%)	24 (27.9%)	0.035
Positive	122 (63.9%)	60 (57.1%)	62 (72.1%)	
VEGF-C				
Negative	50 (26.2%)	36 (34.3%)	14 (16.3%)	0.005
Positive	141 (73.8%)	69 (65.7%)	72 (83.7%)	
Regular shape				
Yes	99 (51.8%)	48 (45.7%)	51 (59.3%)	0.081
No	92 (48.2%)	57 (54.3%)	35 (40.7%)	
Boundary				
Clearly	87 (45.5%)	47 (44.8%)	40 (46.5%)	0.88
Obscure	104 (54.5%)	58 (55.2%)	46 (53.5%)	
Echo				
Homogeneous	85 (44.5%)	46 (43.8%)	39 (45.3%)	0.88
Heterogeneous	106 (55.5%)	59 (56.2%)	47 (54.7%)	
Calcification				
No	95 (49.7%)	46 (43.8%)	49 (57.0%)	0.082
Yes	96 (50.3%)	59 (56.2%)	37 (43.0%)	
Age(years)	49.9 ± 8.9	49.8 ± 8.9	50.0 ± 9.0	0.87
Diameter(cm)	14.6 ± 5.2	12.1 ± 4.2	17.7 ± 4.6	< 0.001
Tumor peak systolic flow velocity(PS)	37.6 ± 6.0	38.2 ± 6.4	37.0 ± 5.5	0.12
Tumor end diastolic flow velocity(EDF)	10.2 ± 2.2	10.2 ± 2.1	10.2 ± 2.3	0.87
Tumor systolic velocity to end of diastolic velocity ratio(SD)	3.9 ± 1.1	3.9 ± 1.0	3.9 ± 1.3	0.36
Tumor resistance index(RI)	0.6 ± 0.2	0.6 ± 0.2	0.6 ± 0.2	0.18
Tumor pulsatility index(PI)	1.8 ± 0.3	1.8 ± 0.3	1.8 ± 0.3	0.55
lymphatic transverse	6.0 ± 1.2	5.9 ± 1.2	6.1 ± 1.3	0.34
lymphatic longitudinal	11.8 ± 2.1	11.8 ± 2.1	11.8 ± 2.0	0.98
cortex area hilum ratio(CH)	1.9 ± 0.3	1.8 ± 0.2	2.0 ± 0.3	< 0.001
Lymph peak systolic flow velocity(PS)	17.4 ± 6.2	15.8 ± 5.7	19.4 ± 6.3	< 0.001
Lymph end diastolic flow velocity(EDF)	7.9 ± 2.1	7.8 ± 1.9	8.0 ± 2.2	0.27
Lymph systolic velocity to end of diastolic velocity ratio(SD)	3.2 ± 1.8	2.4 ± 0.8	4.2 ± 2.1	< 0.001
Lymph resistance index(RI)	0.6 ± 0.2	0.6 ± 0.2	0.6 ± 0.2	0.34
Lymph pulsatility index(PI)	1.7 ± 0.3	1.7 ± 0.3	1.7 ± 0.3	0.25

### Logistic regression and construct predict model

**Fig. 3:**
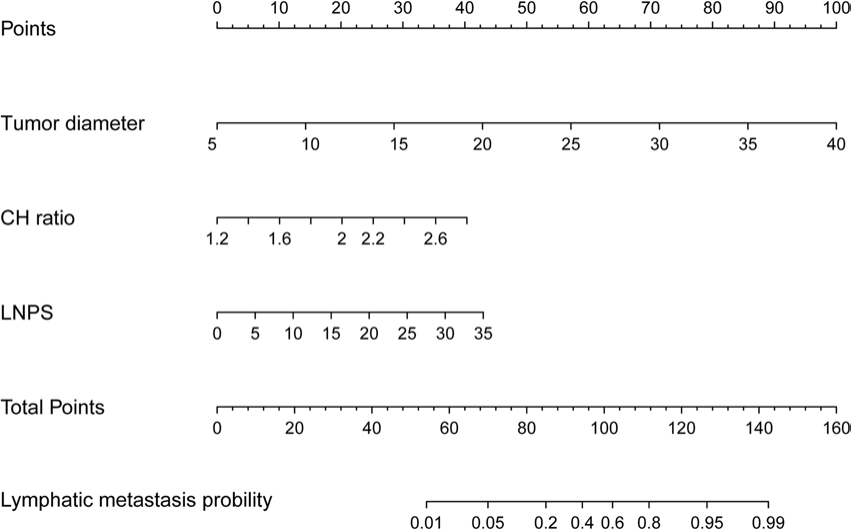
Visualization of the prediction model. If a patient’s tumor diameter is 25 millimeters, corresponding to a score of 58. The CH value is 2, corresponding to a score of 20. The LNPS is 18, corresponding to a score of 18. The total score for this patient is 96, and the probability of lymph node metastasis is 58%, indicating a high-risk group. The pathological results also confirm the presence of lymph node metastasis in this patient

In this study, we adhere to the principles of non-invasiveness and simplicity, selecting only preoperative examination indicators for inclusion in the multifactor analysis. The analysis results are shown in Table 3, the diameter of breast cancer, the PS of lymph, the CH value were enrolled in the logistic regression. Subsequently, based on the analysis results, we constructed a non-invasive predictive model named lymph metastasis predict model (LMPM), and visualized the model, as shown in Fig. 3.

**Table 3:** Logistical result in training cohort

	β	P	OR	OR(95% CI)	
				lower	upper
Tumor diameter	0.297	0.000	1.346	1.219	1.487
Lympha PS	0.128	0.000	1.136	1.065	1.212
Cortexa hilum ratio	2.623	0.000	13.775	3.312	57.285
Contrast	11.886	0.000	0.000		

### Cut-off value of the model

We calculated the risk scores for each patient and utilized the Youden index to find the optimal cutoff value in the modeling cohort, which was determined to be 95 Using this threshold, we divided the patients into high-risk metastasis group and low-risk metastasis group. Subsequently, we computed the predictive indicators of LMPM in both the modeling and validation cohorts, the result showed in Table 4.

**Table 4:** Area under curve of training and validation cohort

	Training cohort			Validation cohort		
Variate	Area under curve	95%CI		Area under curve	95%CI	
		lower	upper		lower	upper
Tumor diameter	0.814	0.754	0.875	0.831	0.733	0.930
Cortexa hilum ratio	0.714	0.639	0.790	0.684	0.565	0.803
Lympha PS	0.670	0.591	0.748	0.701	0.585	0.818
LMPM	0.877	0.829	0.925	0.881	0.801	0.962

### ROC of predict model and other parameters in training and validation cohort

**Fig. 4:**
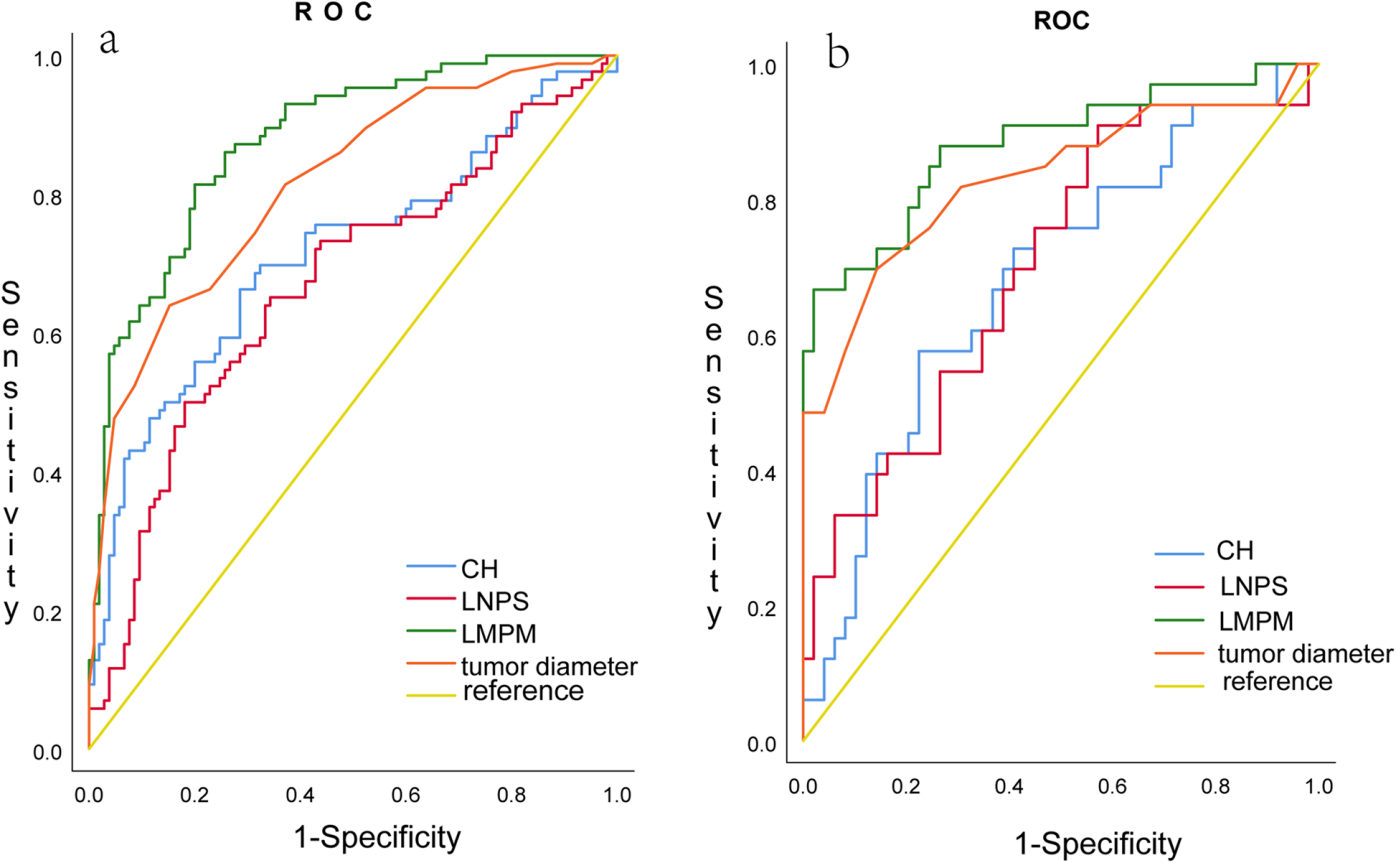
ROC in training and validation cohort. **a** The ROC for different indicators in the training cohort, showed LMPM has larger AUC than other indicators. **b** The ROC for different indicators in the validation cohort, showed LMPM has larger AUC than other indicators

We plotted the ROC curves of LMPM and individual metrics in both the modeling cohort and the validation cohort, as shown in Fig. 4. From the graph, it can be observed that LMPM is located at the far right of the image. Additionally, this study calculated the AUC (Area Under the Curve) for each metric, as presented in Table 5. From the table, it is evident that LMPM has the largest area under the curve, with a value of 0.877(95%CI: 0.829–0.925) in the training cohort and 0.881(95%CI:0.801–0.962) in the validation cohort. Delong test showed that LMPM has larger AUC than PS of lymph and CH value, but not superior than breast cancer diameter, Nonetheless, LMPM still has the largest AUC.

**Table 5:** Evaluations index of training and validation cohort

	Sensitivity	Specificity	Accuracy	Precision	F1score
Training cohort	81.40	76.92	78.94	74.47	77.78
Validation cohort	66.67	97.96	85.37	95.65	78.57

### Calibration curve in training and validation cohort

**Fig. 5:**
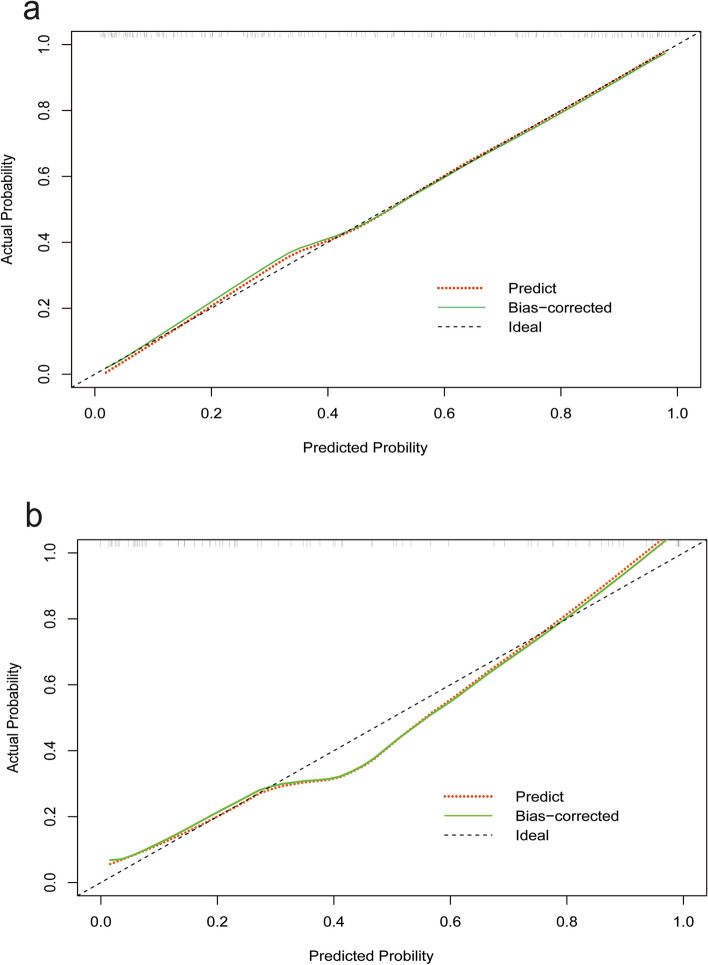
Calibration curve in training and validation cohort. **a** In the training cohort, the predicted curve is distributed along the 45-degree line. **b** In the validation cohort, the predicted curve is distributed around the 45-degree line

We plotted calibration curves in both the training cohort and validation cohort, showed in Fig. 5, and it can be observed that the prediction curves in both groups are distributed around the 45-degree reference line. This indicates that the model we constructed exhibits good accuracy.

### Decision curve analysis (DCA) in training and validation cohort

**Fig. 6:**
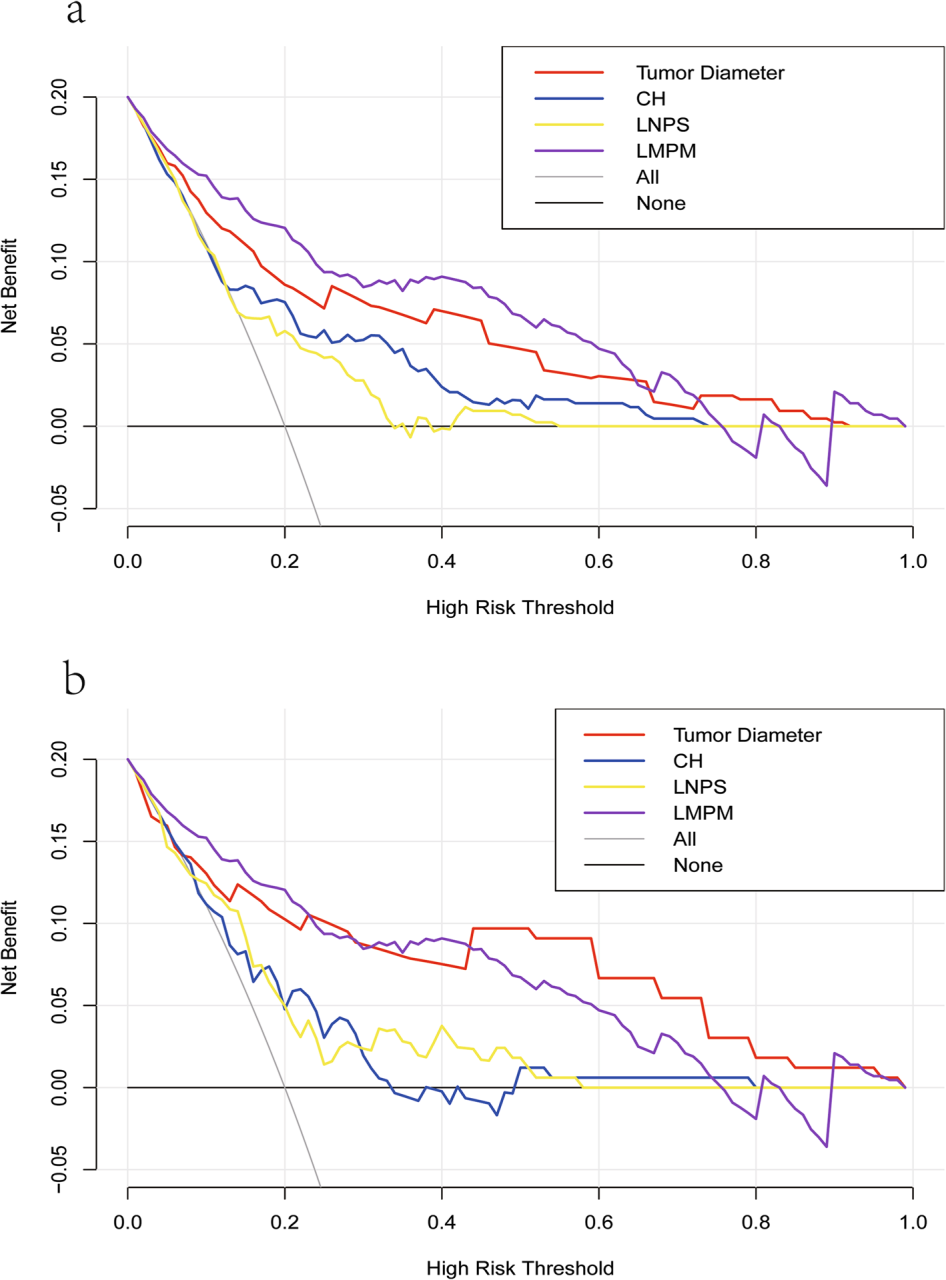
Decision curve analysis in training and validation cohort. The LMPM curve is located in the upper-right quadrant of the graph, indicating optimal clinical decision-making performance. **a** training cohort; **b** validation cohort

We have plotted the DCA curve as shown in Fig. 6. From the graph, it can be observed that LMPM is located in the top right corner compared to other individual indicators, indicating that the model has the best clinical decision-making performance among the other indicators.

## Discussion

The assessment of axillary lymph node status holds significant importance in determining tumor staging, treatment strategies, and prognoses for breast cancer patients. Utilizing non-invasive methods to exclude lymph node metastases can effectively reduce the need for unnecessary axillary surgery. However, relying solely on a physical examination of the axilla is not a reliable approach for predicting lymph node status. Consequently, several imaging techniques have been employed for evaluating axillary lymph nodes. In this study, based on ultrasound examination indicators, a non-invasive prediction model for axillary lymph node metastasis of breast cancer, namely LMPM, was constructed. This model demonstrated good predictive performance in both the training cohort and the validation cohort, with an AUC of 0.877 in the training cohort and an AUC of 0.881 in the validation cohort.

In the past, many scholars have used CT, MRI, nuclear medicine, radiomics, and enhanced imaging methods to predict the metastasis of axillary lymph nodes. However, compared to these methods, ultrasound has been widely accepted by clinical doctors and patients due to its advantages of no radiation, repeatability, and real-time observation. Besides general morphological examinations, ultrasound also has a unique advantage in detecting tiny calcifications within tumors. Additionally, blood flow imaging is a unique advantage of ultrasound examination. Malignant lymph nodes often exhibit specific blood flow characteristics, such as higher blood flow velocity and resistance index compared to benign lymph nodes.

Research has demonstrated the significant impact of cancer size, invasiveness, and lymph node involvement on the prognosis of breast cancer [8]. Larger, invasive tumors with lymph node metastasis exhibit a higher likelihood of recurrence or metastasis. For smaller breast cancers, conservative mastectomy is often the preferred initial treatment option. Effective control of the axillary lymph nodes relies entirely on the lymph node status, whether through extensive dissection or minimal lymph node sampling. In cases where lymph node metastasis is detected, more extensive lymph node dissection becomes necessary. This is why procedures such as sentinel lymph node biopsy or surgical staging of the lymph nodes are frequently recommended. Smaller breast cancers warrant careful consideration due to their lower incidence of lymph node metastasis and reduced invasiveness compared to larger tumors. In this study, for every 1 centimeter increase in the maximum diameter of breast cancer, the risk of lymph node metastasis increased by approximately 34.6%. Consistent with previous studies, larger tumor diameters are associated with a higher risk of lymph node metastasis [9].

When considering blood flow parameter of lymph node, only the parameter PS (peak systolic flow velocity) demonstrated statistical significance in predicting lymph node metastasis. The average PS value observed in our study (19.4 ± 6.3 cm/s vs. 15.8 ± 5.7 cm/s) closely aligned with a previously reported result [10]. This finding is consistent with prior studies that have indicated a correlation between PS and the invasiveness of breast cancer [11]. PS emerged as the most crucial index for predicting lymph node status.

Many scholars focus on studying lymph nodes and believe that the presence of the following signs suggests the existence of lymph node metastasis [12]. These criteria included characteristics such as a round or ovoid shape, a hypoechoic core, a smallest diameter of the node exceeding 5 mm, an irregular cortex, or a cortex thickness greater than 2 mm. However, following the study conducted by [13], the sole criterion for classifying a lymph node as suspicious became a cortex thickness exceeding 2.3 mm. Some scholars also focus on studying the diameter and morphology of lymph nodes as indicators to determine the presence of lymph node metastasis [14]. Sensitivity for node size ranges from 48.8 to 87.1%, while specificity ranges from 55.6 to 97.3%. As for lymph node morphology, sensitivity varies from 26.4 to 75.9%, and specificity ranges from 88.4 to 98.1%.

In order to assess the axillary lymph node status using ultrasound (US), our focus was on identifying cortical changes. Metastatic cells that migrate through afferent lymphatic channels initially settle in the marginal sinus located at the cortex of a lymph node, which eventually drains to the hilum [15, 16]. Cortical thickening serves as an early indicator of metastatic changes. Following cortical enlargement, the subsequent development of a non-fatty hilum is considered a later change and is regarded as the most specific finding for detecting metastases.

To evaluate cortical thickening of a lymph node using ultrasound (US), various methods have been employed, including both quantitative and qualitative approaches. The quantitative indicators encompass measuring the maximum thickness of the cortex, the LT axis ratio, or the number of peripheral blood vessels. On the other hand, the qualitative methods used for diagnosing lymph node metastases through US involve assessing characteristics such as round morphology, hypoechogenicity, loss of central hilum, or eccentric cortical hypertrophy.

Abnormal lymph nodes tend to adopt a more rounded shape due to neoplastic involvement, leading to the enlargement of the short plane of the lymph node [17, 18]. As a result, malignant nodes often exhibit a low LT axis ratio, indicating a round node shape. In Song’s article [19], utilized a cutoff value of 2 since an LT ratio exceeding 2.0 indicates a higher likelihood of the lymph node being non-metastatic [20].

Previous studies investigating cortical thickness on ultrasound (US) have employed different criteria for assessing suspected lymph nodes. A study by Cho et al. [21] proposed a threshold of 2.5 mm for cortical thickness to classify a lymph node as suspicious. To evaluate blood flow within a lymph node, Doppler US, with or without contrast media, has been utilized. Malignant lymph nodes tend to exhibit a higher total number of peripheral vessels compared to benign axillary lymph nodes [22]. However, the variability observed in qualitative or quantitative methods indicates that the results remain inconsistent within each group, as evidenced by meta-analyses [23].

To enhance the diagnostic accuracy of axillary lymph nodes, we conducted measurements of the cortex and hilum areas. We hypothesized that assessing the area might offer greater precision than length alone when evaluating thickened lymph node cortices. By tracing the boundaries of the cortex and hilum on the ultrasound monitor, we obtained the respective areas and compared them. In our current study, we used the concept of “CH” : cortex area hilum ratio, that proposed by Song et.al [19]. Using CH value, we achieved AUC 0.714(95%CI: 0.639–0.790) in training cohort, and 0.684(95%CI:0.565–0.803) in validation cohort.

Our study not only focused on the predictive role of breast cancer diameter in lymph node metastasis but also considered the possibility of overlooking patients with smaller diameters who had already experienced lymph node metastasis if we only considered the diameter. We also investigated the predictive role of tumor and lymph node vascularization in the presence of malignant lymph node metastasis. We found that only lymph node PS (perfusion score) had a predictive effect on lymph node metastasis, with a higher PS indicating a higher probability of lymph node metastasis. Additionally, we included lymph node CH (cortical thickness) in the model, thus taking into account the predictive role of lymph node morphology in lymph node metastasis.

### Limitations

This study has several limitations that should be acknowledged. Firstly, due to the prospective design, we were only able to evaluate a limited number of patients. Specifically, out of the 274 consecutive patients with BI-RADS® category 4B, 4 C, or 5 lesions, only a small number of lymph nodes for analysis. Although the predict model exhibited satisfactory diagnostic performance in distinguishing between metastatic and non-metastatic nodes, it cannot be assumed that it will replace tissue sampling in the near future. Additionally, since the predict model was introduced for the first time in this study, there is no comparable previous research available. Therefore, we recommend conducting further studies with larger populations of breast cancer patients to validate and apply our findings in clinical practice.

Thirdly, we did not conduct an analysis of lymph nodes based on their location or size, which could potentially impact the results and introduce confounding factors. Ultimately, the model’s validation has been limited to internal resampling, leaving its external applicability uncertain. Consequently, it becomes imperative to bolster the model’s reliability by augmenting the sample size through the inclusion of a larger cohort of breast cancer patients. Furthermore, establishing collaborations with external centers for external validation will hold significant importance. Although the prediction model exhibited satisfactory diagnostic performance in distinguishing between metastatic and non-metastatic nodes, the small sample size from a single center makes it difficult to claim the predictions are fully representative for this disease type. This study provides preliminary analysis results that require further validation in larger multicenter trials.

## Conclusion

In summary, we propose the utilization of the predict model as a quantitative parameter for diagnosing lymph node metastasis in axillary lymph nodes using ultrasound. The model demonstrates enhanced diagnostic performance. Further prospective, multicenter studies are needed to validate the practicality of the model.

## Data Availability

The authors declare that all data supporting the findings of this study are available within the paper and its source data for the figures in this study are available from the authors upon request.
